# Supplementary material for: Introducing the trier univalence neutrality ambivalence (TUNA) database: A picture database differentiating complex attitudes
Source: PLoS One. 2024 May 16;19(5):e0302904. doi: 10.1371/journal.pone.0302904 (PMC11098334; doi:10.1371/journal.pone.0302904)
Supplement: S1 Table — (PDF) [file pone.0302904.s001.pdf]

Introducing the Trier Univalence Neutrality Ambivalence (TUNA) Database: A Picture Database Differentiating Complex Attitudes

Table S1

Descriptive Statistics for the total sample as well as the subsamples.

	Total	Representative US	German Students	Convenience	Omnivore	Veg*an	Female	Male
Final *N*	3232	2058	786	388	2771	461	1918	1267
Gender								
Female	1918	1036	630	252	1553	365		
Male	1267	998	154	115	1180	87		
Non-binary	47	24	2	21	38	9		
M_{age} (*SD*)	37.14 (16.72)	44.37 (16.24)	22.05 (3.16)	29.36 (10.49)	38.79 (16.83)	27.24 (12.03)	35.15 (16.61)	40.54 (16.49)
Eating Style								
Omnivore	2771	1962	502	307			1553	1180
Veg*an	461	96	284	81			365	87
M_{BMI} (*SD*)	25.72 (6.64)	28.09 (7.41)	22.45 (3.60)	24.46 (5.38)	26.37 (6.86)	22.74 (4.50)	25.19 (6.67)	26.69 (6.32)
Occupation								
School	39	26		13	34	5	22	17
College/University	1129	216	781	132	806	323	848	266
Apprenticeship	13	8		5	10	3	8	4
Employed	1351	1166	4	181	1254	97	621	710
Pensioner	230	218		12	220	10	120	110
Other	470	424	1	45	447	23	299	160

Note. The validation data is available for the total sample as well as for all subsamples expect the convenience sample.
