# Supplementary material for: Introducing the trier univalence neutrality ambivalence (TUNA) database: A picture database differentiating complex attitudes
Source: PLoS One. 2024 May 16;19(5):e0302904. doi: 10.1371/journal.pone.0302904 (PMC11098334; doi:10.1371/journal.pone.0302904)
Supplement: Attachment — Submitted filename: Response to Reviewers_fin.pdf [file pone.0302904.s002.pdf]

Response to Reviewers

Reviewer #1:

In this study the authors introduce a new picture set and a rating method that enables researchers to differentiate between univalence (positive, negative), neutrality and ambivalence.

The main advantages of the study are (1) the high number of participants; (2) international data collection (US vs. German sample); (3) it provides solution to a methodological problem of previous rating scales.

Although the paper has several strengths, I do not recommend it for acceptance in its current form. Please, find some critics below.
Thank you for the encouraging feedback. We appreciate your detailed comments and believe the manuscript benefitted greatly from them.

R1.1. ABSTRACT
R1.1.1. Affective responses (anger, disgust, empathy) are mentioned in the abstract, but their role and relevance remain unclear in the manuscript. Why did the authors choose these three?

Thank you for pointing this out. Our main goal was to provide a picture database with validation ratings that allow a differentiation between univalence, neutrality, and ambivalence. However, the addition of anger, disgust, and empathy was based on two aspects. First, anger, disgust, and empathy have been shown to be relevant in the food context (Carfora et al., 2021; Kunst & Hohle, 2018; Kunst & Palacios Haugestad, 2016) and second, anger and disgust have been shown to be correlates of ambivalence towards meat (Buttlar et al., 2023; Buttlar & Walther, 2022). However, because our main aim was to generally demonstrate the pitfalls of selecting stimuli based on bipolar valence ratings, for which the three scales are not directly relevant, we report these ratings in the supplemental material (see validation tables; https://osf.io/6br4d/?view_only=e6a303b9d4784c269183ea17eaf54582). In order to clarify the rationality of our design we included the following paragraph into the new version of the manuscript (Page 7-8, Line 156 - 172).

> *"We took several measures to maximize the usability of the TUNA database. First, we aimed to keep the TUNA database compatible with previous databases. Therefore, we oriented our validation on the highly cited food-pics [47] and the FRIDa [32] databases and assessed similar validation ratings including, for example, recognizability, familiarity, desire to eat, palatability, valence, arousal, and complexity. Furthermore and as other databases that include food pictures, the validation tables for the TUNA database include nutritional information for the food images (e.g., [47]) and information about image properties (i.e., color, size, intensity, contrast, complexity; [47]). Second, the TUNA database includes ratings on anger, disgust, and empathy. These affective measures have been shown to be relevant for food consumption [48–50]. Additionally, anger and disgust are correlates of*

*ambivalence toward meat [51,52]. For an analysis of how validation ratings relate, see Buttlar et al. [53]. Third, the database encompasses food pictures in various forms (e.g., raw vs. cooked, non-processed vs. highly processed) because processing has been shown to influence the evaluation of food [48,49]. Fourth, besides the total sample, the validation ratings can be used independent for the representative US and the German student sample. Because research has shown that validation ratings can differ depending on gender [9,47] or diet [47], the validation ratings can also be retrieved for female, male, veg*ans (i.e., vegetarians and vegans) and omnivores separately."*

R1.2. INTRODUCTION

R1.2.1. Page 3, lines 60-61: One of the main focus points of the manuscript is the ambivalence – neutrality difference. If possible, please add some more empirical evidence on the need of differentiation (to demonstrate the gap that the current work fills in).

Thank you for this feedback. We extended the empirical evidence for the consequences of ambivalence and highlighted that only ambivalence and not neutrality should lead to the described consequences. Specifically, we now highlight more detailed examples of ambivalence's affective, cognitive, and behavioral consequences (Page 3 - 4, Line 54 - 70).

> *"Importantly, univalence, neutrality, and ambivalence have fundamentally different affective, cognitive, and behavioral consequences [16]. Affective consequences of ambivalence are, for example, increased negative emotions like uncertainty, anxiety, or irrationality [19]. Additionally, when people experience ambivalence, they also report higher arousal [20]. Because ambivalence elicits these aversive experiences and is related to negative affect [21], it results in other downstream consequences. Cognitive consequences of ambivalence can be biased information processing (e.g., one-sided information search; [22]) or compensatory perception of order (e.g., higher belief in conspiracies; [19]). People also use behavioral strategies to cope with the experience of ambivalence. That is, people who experience ambivalence procrastinate and postpone their decision regarding an ambivalent attitude object [23]. In contrast, neutrality is not related to negative affect and does not instigate those coping strategies, rather it is characterized by a lack of action [17]. We, therefore, argue that ambivalence and neutrality do not only differ conceptually but affect people's responses to stimulus materials. Indeed, previous research indicated differences in the response times and response patterns regarding neutral and ambivalent stimuli [24]. By selecting stimulus material based on valence ratings that cannot account for these differences, researchers may thus draw invalid inferences from their (psychological) studies."*

To highlight this further, we extended the need for differentiation of neutrality and ambivalence in the general discussion (Page 29, Line 649 - 653).

> *"Even for simple categorization tasks (e.g., categorizing an object as positive vs. negative), there are response differences between ambivalence and neutrality. That is, responses to ambivalent stimuli are significantly faster than responses to neutral stimuli [24]. Thus, selecting supposedly neutral stimulus material based on bipolar*

> *valence ratings might lead to unwanted effects when inadvertently ambivalent material was selected."*

R1.2.2. P 6, ls119-127: It is unclear, if the intention was to develop a food-specific dataset? Why were non-food pictures included? What is the reason behind to pick pictures from these categories? Are food pictures and non-food pictures relate to eachother (e.g. through associations, context, cultural aspects etc)?

Thank you for this question. Even though the TUNA database includes many pictures of food, it was not supposed to be solely a food picture database. Our overarching goal was to create a database that encompasses a diverse array of attitude objects. Due to the inherent complexity of ambivalent attitudes, capturing them unequivocally in a photograph presents challenges. In previous research the stimuli were presented as words (e.g., organ donation, abortion, drug use). However, unambiguously depicting these attitude objects in a picture is difficult. For instance, a photograph of an injection needle could potentially signify drug use (i.e., an ambivalent attitude object) or convey the administration of life-saving medication (i.e., a univalent positive attitude object). Hence, capturing some previously used ambivalent attitudes in a visual form proved to be challenging, whereas depictions of ambivalent food categories like unhealthy foods or meat were readily achievable. Additionally, it was feasible to include univalent (i.e., healthy food) and neutral (i.e., carbs) food pictures. Beyond food-related content, our efforts extended to incorporate other ambivalent objects like vehicles or money, along with pictures of non-food related univalent (e.g., flowers) and neutral (e.g., toaster) objects.

We now explain our rationale for the picture selection (Page 6 – 7, Line 133 - 155).

> *"A challenge for the creation of the TUNA database was finding picture stimuli that are ambivalent. Previous ambivalence research predominantly used word stimuli (e.g., questionnaires; [33–35]). This might be due to the fact that ambivalent attitude objects like abortion [36], drug use [34], physical exercise [37], organ donation [38], or romantic partners [33], are difficult to unambiguously capture in photographs (e.g., does an injection needle signify drug use or a life-saving medication injection). However, some ambivalence research has utilized picture stimuli (e.g., [25,31,39–43]). These studies predominantly focus on attitudes toward food [25,31,39,40,42]. Even when the emphasis is not on food, the picture may still feature food-related elements (e.g., plastic-packed or unpacked food when investigating plastic related ambivalence; [41]) or an explanation of the picture was provided before the evaluation of the pictures [43]. The rationale behind this food-centric focus lies in the distinctiveness of food as a source of particularly salient univalent (e.g., fruits; [25]), neutral (e.g., bread or oats; [44]), and ambivalent (e.g., meat or chocolate; [35,45]) attitudes.*
>
> *While the majority of images in the TUNA database portray food, it also includes a diverse array of potentially ambivalent attitude objects. For example, in the study exploring ambivalence in the IAPS pictures, various attitude objects such as a hammer, wolf, chair, and mug elicited ambivalence [20]. Consequently, the TUNA database also encompasses pictures of potentially ambivalent items (e.g., hammer, mug, chair) and animals (e.g., dog, cow) as well as other ambivalent content like vehicles or money. Because ambivalence research oftentimes incorporates univalent*

> *and/or neutral control categories (e.g., [24,31]), the TUNA database additionally includes pictures of attitude objects that are typically used in research to study univalent (e.g., flowers) and neutral (e.g., toaster) attitudes [7,9,46,47]. Hence, the TUNA database encompasses a variety of univalent, neutral, and ambivalent picture stimuli."*

Additionally, in the method section, we state that the picture selection was based on what feasibility (Page 10, Line 217 - 220).

> *"Because all pictures of the TUNA database were exclusively photographed for this purpose, the content is restricted to what was feasible to photograph. However, we tried to include various univalent, neutral, and ambivalent attitude objects to heighten the usability of the database."*

Furthermore, we acknowledge possible limitations that might result based on the stimulus selection in the discussion (Page 31 – 32, Line 706 - 718).

> *"Another limitation of the TUNA database pertains to its stimuli, primarily comprising food images. This bias stems from the ease of depicting food and its clear categorization into univalent, neutral, and ambivalent conditions. Using these stimuli, we demonstrated the necessity to differentiate univalence, neutrality, and ambivalence with food stimuli. Even for non-food stimuli incorporated in our database, such as money, vehicles, household items, or flowers, we would expect the same results. Validation ratings measured via split semantic scales, but not bipolar scales should enable researchers to differentiate between neutrality and ambivalence. While researchers might not select a wide variety of non-food stimuli from the database as picture content was limited based on feasibility, the chosen approach also resulted in adopting a CC BY-NC-SA 4.0 copyright. Hence, researchers can still select various univalent, ambivalent, and neutral pictures from the TUNA database which can be shared and adapted for non-commercial use when the original authors are credited, and the adaptations are shared under the same terms."*

R1.2.3. I'm not sure if all the information in paragraph #2 (p 6, from line 128) should be here. The number of validation ratings, participants and self-report/picture are the results of the validation process, while information in ls 135-138 are demonstarting the goals of the study. Please, modify it or find its place at another part of the manuscript as a conclusion.

Thank you for this comment. We restructured the paragraph so that it now summarizes the content of the TUNA database (Page 8- 9, Line 173 - 190).

> *"In sum, the TUNA database is a highly versatile picture database. The TUNA database encompasses various validation ratings (i.e., positivity and negativity, which were also combined into a potential ambivalence index, felt ambivalence, valence, arousal, anger, disgust, empathy, palatability, desire to eat, familiarity, recognizability, and complexity), objective image characteristics (i.e., color, size, intensity, within-object contrast, and object complexity) as well as nutritional information for food pictures (i.e., protein, carbs, fat, and kcal per 100g). This extensive*

> *range of information allows researchers to consider various facets of human stimulus perception and (emotional) response when selecting their study material. Additionally, the total of 304,262 validation ratings were from a heterogeneous sample of 3,232 participants, and at least 20 ratings per self-report scale per picture were collected. However, to account for sample specifics and enable researchers with a specific target sample, the validation data can be retrieved for the different subsamples (i.e., German student, representative US, male, female, veg*ans, omnivores). Furthermore, to maximize accessibility and streamline the research process, the TUNA database is accompanied by a user-friendly access tool (https://osf.io/fys36/?view_only=48a9c56e7d264285b9d053369e3de15d). This tool facilitates the selection of suitable stimulus material based on the extensive validation ratings. Hence, making the TUNA database an invaluable asset for researchers seeking a comprehensive resource for picture stimuli."*

R1.2.4. P7, l 140: Please, clarify the reason why these three emotions were selected!

Thank you for this feedback! Please see our response to your comment R1.1.1.

R1.3. METHODS

R1.3.1. P 8, from l 169: The number of pictures in each category are unequal, which is not a problem in itself, but it is unclear what was the conceptual background of selection? Why vegetable pictures are so dominant? What is the ratio of food and non-food pictures? (p 15, from l 328 might be the answer…)

Thanks for the comment! We extended the explanation that the database should be as versatile as possible and that the picture selection was based on what was possible to photograph. See also our response to your comment R 1.2.2.

R1.3.2. P 9, ls 187-188: Please, explain the relevance of adding protein, carb etc. Information of food-pictures? (If there is another conceptual reason of the study that makes it relevant to include these nutrition details, why was it necessary to add non-food pictures to the database?)

Thank you for this question. The nutritional information was added to make the TUNA database compatible with the food-pic database from Blechert et al. (2014). As mentioned in our response to your first comment (see R1.1.1), we wanted to increase the usability of the database and tried to make the database compatible with other databases. Going beyond the changes indicated in response to your first comment, we also state in the method section that the nutritional information was added to make the TUNA database compatible with the food-pics database (Page 10, Line 237-238).

> *"For all food pictures, protein, carbs, fat, and kcal per 100g are included in the validation table. This allows comparability of the TUNA and the Food-Pics [47] database."*

R1.3.3. P 10, ls 228-232: Without a clear theoretical background in the

Introduction, it is hard to see why anger, disgust and empathy ratings are collected. Please, clarify it.

Thank you for this feedback. Please see our response to comment R.1.1.1.

R1.3.4. If I understood it correctly, the convenience sample consisted of some US and some German particiapnts. As the US and German sample do have some significant differences (see Table 1.) it might be the case, that this third group shows the mean of the first two samples. From the results it seems to me that the US and German samples have different eating styles, preferences, BMI etc. It looks like a culture-specific aspect.

Thank you for this observation. We performed all analysis for the representative US and German student sample separately. As with the analyses for the total sample, we calculated all analyses three times with 50 randomly drawn pictures from the ambivalent meat and univalent fruit category. Specifically, we calculated one-factor (category: univalent positive fruit vs. neutral carbs vs. ambivalent meat vs. ambivalent sweets) ANOVAs with potential ambivalence, valence, positivity, negativity, felt ambivalence and arousal as dependent variable. Descriptively, all effects are in the expected direction, however, due to lower power some Bonferroni corrected pairwise comparisons do not reach significance. In sum, the results of the analysis show that a bipolar valence scale cannot differentiate between univalence, neutrality, and ambivalence, and these different attitudes lead to different levels of experienced conflict and arousal. The results of the separate multinomial logistic regressions for the representative US and German student sample are also consistent with the results from the total sample. That is, the test model including potential ambivalence as an additional predictor always explains significantly more variance than the baseline model that only includes valence as predictor. Additionally, the results consistently show that the proportion of correct classified pictures increases when potential ambivalence is added as predictor. Hence, the separate analyses for the representative US and German student sample also highlight the need to differentiate univalence, neutrality, and ambivalence when selecting study material to allow unambiguous interpretation of the study results. Because the results of the separate analyses have such high overlap with the results of the total sample, we only summarize the results in the main text (Page 26 - 27, Line 582 - 605), however, we report all analyses and results in detail in the supplemental material.

> *"**Analyses for the representative US and German student sample***
>
> *We also performed all analyses for the representative US and German student sample separately. Hence, we calculated one-factor (category: univalent positive fruit vs. neutral carbs vs. ambivalent meat vs. ambivalent sweets) ANOVAs with potential ambivalence, valence, positivity, negativity, felt ambivalence, and arousal as dependent variables for the representative US and the German student sample. Consistent with the results for the total sample, we found that univalent positive fruits had the lowest potential ambivalence, followed by neutral carbs. The ambivalent categories had the highest potential ambivalence. Additionally, and as expected, the bipolar valence ratings were insufficient to distinguish univalence, neutrality, and ambivalence. As for the consequences of ambivalence, the results show that the ambivalent categories elicit higher felt ambivalence and higher arousal than the univalent and neutral category. Descriptively, all effects are in the expected direction,*

however, due to lower measurement accuracy and power in the subsamples, not all Bonferroni corrected pairwise comparisons reached significance. However, the results consistently show that a bipolar valence scale cannot differentiate between univalence, neutrality, and ambivalence. Furthermore, the results show that these different attitudes lead to different downstream consequences, which can affect how people respond to the pictures.

We also performed multinomial logistic regression analyses for both subsamples separately. The results show the superiority of the test model, including valence and potential ambivalence as predictors. Additionally, adding potential ambivalence as a predictor improves the proportion of correctly categorized pictures (see supplemental material for details https://osf.io/8pj94/?view_only=3ce87d481c5d42008e98e1771f1e156b). In sum, the separate analyses for the representative US and German student sample replicate the results of the total sample."

Additionally, we revised the paragraph about the limitation of cross-cultural generalization in light of these results (Page 32, Line 719 - 735).

*"A third possible limitation of the TUNA database might be the samples used for validation. Even though the separate analyses for the German student and representative US sample replicate the results of the total sample and highlight that a bipolar valence scale is insufficient to differentiate complex attitudes, there are minor (descriptive) differences in evaluation extremity (see https://osf.io/8pj94/?view_only=3ce87d481c5d42008e98e1771f1e156b). For example, whereas the ambivalent meat category has higher potential ambivalence than the ambivalent sweets category in the representative US sample, the ambivalent sweets category has higher potential ambivalence than the ambivalent meat category in the German student sample. The divergence between samples might indicate some sample specific aspects that influence the evaluation of the pictures. Such differences might be the higher proportion of veg*ans in the German student sample compared to the representative US sample or the higher BMI in the representative US sample compared to the German student sample. Because Germany and the USA are Western, Educated, Industrialized, Rich, and Democratic (WEIRD; [57]) and the overall effects of our analyses are the same, however, we argue that, it is possible to use the combined ratings. However, the cross-cultural generalization is limited, and further research is needed to confirm the database's validity in other samples."*

R1.3.5. P 14, from line 297: the procedure is clear for the German sample. Was it the same in the US sample, as well?

Thank you for pointing this out. We revised the section and clarified that the procedure was the same except for the explicitly mentioned deviations (Page 16, Line 346 - 348).

"Data collection differed slightly across the different samples. In the following, we describe the deviations from the procedure, if not stated otherwise, the procedure was the same as described in the section on procedure and data collection."

R1.3.6. P 14, l 302: I suggest to leave out the Meat Ambivalent Questionnaire and the other study. It makes difficult to follow the logic of the current manuscript.

Thank you for this suggestion. We understand your concern and acknowledge that this might raise questions among readers. However, it might raise even more critical questions if we would not describe such deviations in the procedure for the different samples. For instance, if we only describe that participants answered the Meat Ambivalence Questionnaire and that the pictures were not randomly drawn, this might make readers wonder why we deviated from the completely randomized picture selection used in the other studies. We would have preferred to give this information in a footnote to help the logic of the manuscript. However, Plos ONE does not permit footnotes. Therefore, we keep the three sentences regarding validating the Meat Ambivalence Questionnaire.

R1.3.7. 9 14, l 308: What was the attention-check question exactly?

Thank you for bringing this to our attention. We added the exact wording of the attention check question (Page 16 - 17, Line 367 - 370).

> *"In the prolific and German student survey, there were also two attention-checks. The attention-checks were two instructed response questions ("This is a check of your attention: Please move the slider completely to the right [left] i.e., "agree" ["disagree"]") and were randomly intermixed with the other question blocks."*

R1.4. RESULTS
R1.4.1. P 15, l 315-316: Please, clarify this sentence.

Thank you for pointing out this unclarity. We revised this sentence (Page 19, Line 425 - 426).

> *"Each of the 796 pictures was rated at least 20 and maximally 45 times (M = 27.3, SD = 4.84)."*

R1.4.2. If the distinction of food and non-food pictures is important, please provide the identifiability according to the sub-categories in the dataset.

Thank you for this suggestion. We do not believe that the sub-categories are essential for the manuscript besides highlighting the versatility of the TUNA database. However, we now provide the mean for the pictures used in the analysis in the text (Page 20, Line 449 – 453) and Table 2 (Page 21).

> *"The lowest mean for recognizability was 58.42, indicating that, on average, pictures were categorized more often as easy than difficult to recognize, and the lowest mean for familiarity was 74.94, indicating that, on average, the pictures were categorized more often as familiar than unfamiliar. Hence, the average identifiability of the categories was sufficient for the analysis."*

R1.4.3. P15, l 328-332: Please, clarify the replication aim with previous research more detailed.

Thank you for pointing this out. We now clarified what we tried to replicate (Page 17 - 19). Consistent with your next comment, we therefore included an 'Expectations' section stating the hypothesis and replication aim (see our response to R1.4.4.).

R1.4.4. P 16, from l 346: It would be more elegant to include predictions/expectations/hypotheses before the results section.

Thank you for this feedback. We included an 'Expectations' section describing the hypotheses and the replication aim (Page 17 – 19, Line 375 - 422).

*"**Expectations***

By validating the TUNA database, we aimed to provide a standard set of pictures, including various attitude objects that can be distinguished based on more complex attitudes: univalence, neutrality, and ambivalence. To demonstrate the importance of distinguishing complex attitudes, we provide a case study using stimuli that have previously been assumed to be univalent, neutral, or ambivalent. Specifically, it has been argued that fruits are univalent positive [25], carbs like bread or oats are neutral [44] and sweets [25] as well as meat [45] are ambivalent. Our aim was to demonstrate that the validation ratings within the TUNA database align with the anticipated attitudes on split semantic scales, indicating potential ambivalence, but not on bipolar valence ratings. Furthermore, our intention was to illustrate that these variations in attitudes could yield substantial implications if researchers fail to account for them while selecting stimuli.

In our first analysis, we expected that the ambivalent categories (sweets and meat) have higher potential ambivalence than the univalent (fruits) and neutral (carbs) categories. We additionally expected that the neutral category has higher potential ambivalence than the univalent category because negative values on the SIM-Index as an indicator of potential ambivalence suggest univalence, and values around zero suggest neutrality [20]. Hence, we would first replicate that the univalent, neutral, and ambivalent categories used in previous studies are indeed univalent, neutral, and ambivalent [25,44,45].

In addition, we wanted to show that bipolar valence scales might not capture these differences in attitudes. While ambivalent attitude objects typically elicit a rating in the middle of a bipolar valence scale as if they were neutral [20], other ambivalent attitude objects elicit rather positive ratings. For instance, Norris et al. [25] demonstrated that unhealthy food (e.g., sweets) and healthy food (e.g., fruits) have similarly positive ratings on a bipolar valence scale, although, they differ on potential ambivalence when measured via split semantic scales. We, therefore, expected that fruits would not differ from sweets on a bipolar valence scale, and we expected that carbs and meat do not differ on a bipolar valence scale. However, on the same scale, we expect fruits and sweets to have higher valence ratings than carbs or meat. This would demonstrate that bipolar valence scales cannot distinguish between complex attitudes.

Then, we aimed to highlight the added value of potential ambivalence for predicting category membership of the pictures. Using multinomial logistic regression with neutral carbs as reference category, we expect that the prediction for category membership improves when potential ambivalence is added as an additional predictor compared to a baseline model, including only valence measured with a bipolar scale as a predictor. Hence, we expect that the test model, including potential ambivalence as a predictor, explains more variance and has a higher percentage of correctly categorized pictures than the baseline model. Specifically, the superiority of the test model means that taking potential ambivalence into account reduces the confusion between ambivalent and neutral as well as univalent and neutral stimuli. This would reveal that potential ambivalence should be considered when selecting univalent, neutral, and ambivalent stimuli.

Finally, we wanted to demonstrate that univalence, neutrality and ambivalence not only differ in their structure of positive and negative associations but are also associated with different affective responses. Specifically, we expect the ambivalent categories to have higher felt ambivalence and arousal than the neutral and univalent categories [20,23]. The neutral and univalent categories should not differ in their felt ambivalence and arousal. This would highlight that researchers who solely rely on bipolar valence ratings to select stimulus material may overlook significant differences within people's attitudes that can distort the perception of the stimuli."

R1.4.5. p 17, ls 374-375: this results are really important! In my opinion, this is key point of the manuscript.

Thank you for pointing this out. We fully agree with your assessment and extended the description of the result (Page 22, Line 486 - 491).

"Thus, results indicate that a bipolar valence scale cannot differentiate between univalent, neutral, and ambivalent pictures. Additionally, the finding that mere valence is insufficient to differentiate univalence, neutrality, and ambivalence is consistent even when the German student and representative US sample are analyzed separately. This highlights that when selecting stimulus material based on a bipolar valence scale, researchers might inadvertently select ambivalent material instead of univalent or neutral material."

R1.4.6. Results in Table 2 and Table 3 were calculated from the total sample? I wonder if the results are the same in the US and German samples, respectively? (Based on the differences in Table 1, they should be.)

Thank you. We calculated the separate analyses, which converge. The results support the main point of the manuscript. That is, a bipolar valence scale is insufficient to differentiate univalence, neutrality, and ambivalence. There are minor (descriptive) differences between the samples in attitude extremity. For example, in the German student sample sweets have higher potential ambivalence than meat whereas meat has higher potential ambivalence than sweets in the representative US sample. We acknowledge this in the limitation section. However, because the effects of interest (i.e., the ambivalent categories eliciting higher

ambivalence than the univalent and neutral category, bipolar valence scale cannot differentiate complex attitudes and adding potential ambivalence as predictor of attitude categorization improving the model as well as increasing the proportion of correctly classified pictures) are all in the expected direction and replicate the total sample, we only summarize these results in the main text. All results and the tables for these analyses are presented in detail in the supplemental material (https://osf.io/8pj94/?view_only=3ce87d481c5d42008e98e1771f1e156b; please also see our response to R1.3.4.).

R1.5. DISCUSSION

R1.5.1. Please, consider to keep the focus of the manuscript only on food material and sub-categories such as meet, fruits, bread, oats etc. (in accordance with p 22, l 478 'focus on food').

Thank you for this comment! We realize that the database comprises many food pictures and that our analyses are also within the food domain. However, the analyses are meant to demonstrate why it is crucial to distinguish univalence, neutrality, and ambivalence. Going beyond, we argue that the importance of differentiating complex attitudes also holds in non-food contexts. In fact, based on the non-food pictures within the TUNA database, researchers can select stimuli based on our validation ratings outside of the food domain. We believe that focusing solely on food might discourage some researchers from doing so, and, therefore, we would like to keep the paper in its current form. Nonetheless, we now acknowledge the high prevalence of food pictures within the manuscript on page (see also our response to your comment R1.2.2.).

R1.5.2. Maybe the TUNA App should be the topic of a different publication.
Thank you for this suggestion. Even though presenting the TUNA desktop app in a different publication might have advantages, we believe that keeping all key information about the TUNA database and how to use it in the same manuscript will facilitate the use of the database. However, to avoid confusion of the TUNA app with an app for a mobile phone, that might suggest an independent use, we now consistently refer to it as desktop app. Additionally and to highlight the usability of this easy-to-use tool, we changed the part introducing the desktop app in the manuscript (Page 30, Line 669 - 685).

> ***"How to access TUNA***
>
> *Even though the validation tables and pictures are accessible via the supplemental material, we recommend accessing the TUNA database via its accompanying desktop app. This tool is designed to facilitate stimulus selection while simultaneously accounting for the complexity of attitudes, compiling all validation data and images into a unified location. In the first tab, a description of the database and desktop app is presented. In the sidebar, the filter options are located. All filters are preset so that all validation ratings for the total sample are displayed. However, changing a filter option leads to a real-time adaptation of the validation tables. All self-report scales can be used to filter the pictures. These filter options make it easy to exclude pictures,*

for example, due to lower recognizability. The picture selection resulting from the currently set filters and the associated validation table can be downloaded via the buttons in the bottom left corner (i.e., Download Data or Download Images). This facilitates comparison of picture selection based on different subsamples. Besides the data table with all variables (Full Data Table), the data table with only the self-reported ratings (Self-Reported Variables) or with only the picture characteristics (Image Characteristics) can be selected via the tab menu. In the table, a thumbnail of the picture is displayed beside the validation ratings (see Fig 2)."

R1.5.3. P 25, l 555: How is the 'cultural background' defined? Why are the same?
Thank you for pointing out this ambiguity. We referred to this expression because both samples come from so-called WEIRD populations (Henrich et al., 2010). We now clarified this in the manuscript and have additionally revised this paragraph in light of the results of the separate analysis of the representative US and German student sample (Page 32, Line 719 - 735; see also our response to your comment R1.3.4.).

In sum, I do not suggest the manuscript for acceptance in its current form due to some conceptual and methodological concerns. Please, consider (1) if you wish to keep the focus on food pictures only and (2) to investigate the effects in the US and German samples separately.

Thank you for your helpful feedback. We believe that this helped us immensely to improve the manuscript.

As for the first point, the main aim of the database was to provide validated pictures that can be differentiated as either univalent, neutral, or ambivalent attitude objects. While we argue that this aim is independent of the domain, we now stress at several points of the manuscript that the majority of pictures are food pictures and why this is the case. We also highlight that our analyses are within this domain (see our response to your comments R1.2.2. and R1.5.1.).

As for the second point, we present the separate analysis of the US and German samples in detail in the supplemental material and summarize the results in the 'Analyses for the representative US and German student sample' section in the main text. The analyses converge. That is, all analyses indicate that bipolar valence ratings cannot distinguish complex attitudes and using potential ambivalence ratings to differentiate univalence, neutrality, and ambivalence improves correct attitude categorization (see our response to R1.3.4/1.4.6.). Thus, we decided to stick to the analyses with the combined samples for the main text and present the separate analysis in the supplemental material. However, we discuss possible limitations due to the sample and derive recommendations for the stimuli selection from the TUNA database (see our response to R1.5.2.).

Reviewer #2:

This is a normative study about the affective (an other) properties of a large set of pictures. Its greatest contribution is to assess affectivity, not using a bipolar scale, but unipolar scales of positivity and negativity. The analyses performed demonstrate that using bipolar scales is not the proper procedure for distinguishing neutrality from ambivalence. This has important implications for research on affective processing (which commonly relies on normative datasets), evidencing the need to be very careful in the selection of "neutral" stimuli. Therefore, the paper is a good and useful contribution to the field and deserves to be published. I have, however, some comments that the authors need to consider before the paper is accepted. I list them below:

Thank you for reviewing our manuscript. We appreciate your feedback and comments. We believe that they have helped improve the manuscript's quality even further.

R2.1. Introduction

R2.1.1. -The normative study is mostly focused on food pictures. The authors need to provide a justification for this choice (which may limit the applicability of the dataset).

Thank you for this comment. Indeed, a majority of the pictures come from the domain of food. In line with the request of Reviewer #1, we now included an explanation for the stimulus selection which was driven by feasibility (Page 10, Line 217-220). Additionally, and consistent with your suggestion, we now include possible limitations due to the available pictures in the TUNA database (see comment R1.2.2. for the implemented changes).

In the Introduction, we now explain why most pictures are of food (Page 6 – 7, Line 133 - 155).

> *"A challenge for the creation of the TUNA database was finding picture stimuli that are ambivalent. Previous ambivalence research predominantly used word stimuli (e.g., questionnaires; [33–35]). This might be due to the fact that ambivalent attitude objects like abortion [36], drug use [34], physical exercise [37], organ donation [38], or romantic partners [33], are difficult to unambiguously capture in photographs (e.g., does an injection needle signify drug use or a life-saving medication injection). However, some ambivalence research has utilized picture stimuli (e.g., [25,31,39–43]). These studies predominantly focus on attitudes toward food [25,31,39,40,42]. Even when the emphasis is not on food, the picture may still feature food-related elements (e.g., plastic-packed or unpacked food when investigating plastic related ambivalence; [41]) or an explanation of the picture was provided before the evaluation of the pictures [43]. The rationale behind this food-centric focus lies in the distinctiveness of food as a source of particularly salient univalent (e.g., fruits; [25]), neutral (e.g., bread or oats; [44]), and ambivalent (e.g., meat or chocolate; [35,45]) attitudes.*
>
> *While the majority of images in the TUNA database portray food, it also includes a diverse array of potentially ambivalent attitude objects. For example, in the study exploring ambivalence in the IAPS pictures, various attitude objects such as a hammer, wolf, chair, and mug elicited ambivalence [20]. Consequently, the TUNA database also encompasses pictures of potentially ambivalent items (e.g., hammer, mug, chair) and animals (e.g., dog, cow) as well as other ambivalent content like vehicles or money. Because ambivalence research oftentimes incorporates univalent*

and/or neutral control categories (e.g., [24,31]), the TUNA database additionally includes pictures of attitude objects that are typically used in research to study univalent (e.g., flowers) and neutral (e.g., toaster) attitudes [7,9,46,47]. Hence, the TUNA database encompasses a variety of univalent, neutral, and ambivalent picture stimuli"

In the Method section, we added that the picture selection was partly due to available objects to photograph (Page 10, Line 217 - 220).

"Because all pictures of the TUNA database were exclusively photographed for this purpose, the content is restricted to what was available to photograph. However, we tried to include various univalent, neutral, and ambivalent attitude objects to heighten the usability of the database."

Furthermore, as you suggested, we added a paragraph about how the applicability might be limited due to the stimuli in the discussion (Page 31 – 32, Line 706 - 718).

"Another limitation of the TUNA database pertains to its stimuli, primarily comprising food images. This bias stems from the ease of depicting food and its clear categorization into univalent, neutral, and ambivalent conditions. Using these stimuli, we demonstrated the necessity to differentiate univalence, neutrality, and ambivalence with food stimuli. Even for non-food stimuli incorporated in our database, such as money, vehicles, household items, or flowers, we would expect the same results. Validation ratings measured via split semantic scales, but not bipolar scales should enable researchers to differentiate between neutrality and ambivalence. While researchers might not select a wide variety of non-food stimuli from the database as picture content was limited based on feasibility, the chosen approach also resulted in adopting a CC BY-NC-SA 4.0 copyright. Hence, researchers can still select various univalent, ambivalent, and neutral pictures from the TUNA database which can be shared and adapted for non-commercial use when the original authors are credited, and the adaptations are shared under the same terms."

R2.1.2. -The normative study includes ratings of anger, disgust and empathy. There is no justification for the inclusion of these variables/emotions and not others (e.g., fear, sadness, happiness). The authors should explain the relevance of these variables.

Thank you for this feedback. We now describe why we chose these validation ratings (Page 8, Line 163 - 166; see our response to the comment of Reviewer #1: R1.1.1.). Specifically, anger, disgust, and empathy have been shown to be relevant for food consumption (Carfora et al., 2021; Kunst & Hohle, 2018; Kunst & Palacios Haugestad, 2016) and anger as well as disgust are correlates of ambivalence towards meat (Buttlar et al., 2023; Buttlar & Walther, 2022). Because the focus of our manuscript is not solely on food, we do not use these variables. However, we now justify the selection of these variables in the Introduction.

"Second, the TUNA database includes ratings on anger, disgust, and empathy. These affective measures have been shown to be relevant for food consumption [48–50]. Additionally, anger and disgust are correlates of ambivalence toward meat [51,52]. For an analysis of how validation ratings relate, see Buttlar et al. [53]."

R2.2. Methods and analyses

R2.2.1. -As far as I understand, each participant evaluated a small set of pictures in several variables. The order of the variables was random, except for valence, positivity, negativity and felt ambivalence, which were rated always in the same order. The authors should justify this procedure/order. The assessment of felt ambivalence may have been affected by the previous assessment of the other variables.

Thank you for this feedback. The order of the items is based on the model of ambivalence induced discomfort (van Harreveld et al., 2009), which states that potential ambivalence is a prerequisite for felt ambivalence. We now justify the order in the manuscript (Page 12 - 13, Line 286 - 298).

> *"The questions to differentiate between attitudes (e.g., neutrality and ambivalence) were presented in a fixed attitude question block. Valence (Evaluate this object, scale: very negative to very positive) was always followed by positivity (How positive is this object regardless of its negative aspects?, scale: not positive at all to very positive) and negativity (How negative is this object regardless of its positive aspects?, scale: not negative at all to very negative), which in turn were followed by felt ambivalence (To what extent do you experience conflicting thoughts or feelings towards this object?, scale: not at all conflicted to maximally conflicted). These split semantic scales on positivity and negativity were used to calculate a potential ambivalence score based on the SIM-Index [(Positivity + Negativity)/2 - |Positivity – Negativity|], where higher values stand for more ambivalence, lower values for univalence, and values around zero for neutrality [20]. Note that we assessed potential ambivalence first because potential ambivalence is considered to be a prerequisite of felt ambivalence [23]. The attitude question block was randomly presented with the other questions."*

R2.2.2. -Was a definition of "emotional arousal" provided to participants? If not, it must have been difficult for them to rate this variable. This may have produced a large variability in the ratings.

Thank you for this feedback. We extended the description (Page 12, Line 269 - 273). Before each question block, a brief description of the following question was provided.

> *"Before each block of questions, participants read a brief introduction for the next construct. For example, before answering the arousal question, participants read the following instruction: "In the following, you are going to be asked in what way this object triggers an emotional arousal in you. Please move the slider spontaneously to the position that best describes your current assessment."*

Even though the participants did not receive a definition of "emotional arousal", we believe that the participants were able to rate this variable. The SDs do not indicate larger variability in the arousal rating. See, for example, Table 2. The SDs for the categories of arousal ranged from 7.17 to 8.11. The SDs from all other scales with the same range (i.e., Valence, Positivity, Negativity, Felt Ambivalence) ranged from 5.97 (Felt Ambivalence in the

ambivalent meat category) to 16.51 (valence in the univalent positive fruits). Hence, the variability of the arousal ratings is not greater than for other ratings with the same scale.

R2.2.3. -Although the dataset is focused on food-related images, it also includes other types of pictures. There are probably univalent, ambivalent and neutral images among them. The authors should perform the same analyses that they have done with food-related images, with these other non-food images. This would inform the readers about the generalizability of the obtained results.

Thank you for this feedback. As outlined in response to Reviewer #1 (see R1.2.2.), many ambivalent attitude objects are abstract constructs and not easily depictable (e.g., organ donation, romantic relationship). Thus, a majority of pictures came from the domain of food. Nonetheless, we tried to add pictures for other ambivalent categories, such as money or cars, to increase the usability of the database. Unfortunately, the number of pictures from these categories is quite low (n_{Money} = 12; $n_{Vehicles}$ = 6). Therefore, the pictures were not rated often enough to achieve adequate power for similar analyses within non-food domains. Because we only ran the analyses on the food pictures only to demonstrate the importance and benefits of distinguishing between univalence, neutrality, and ambivalence, this does not limit the use of the validated non-food pictures for future research. Nonetheless, we now highlight that this focus on food pictures is a possible limitation within the general discussion (Page 31 – 32, Line 706 - 718; see our response to your comment R2.1.1.).

R2.2.4. -Why were ratings of disgust, anger and empathy collected if no analysis was performed on these data?

Thank you for your question regarding the inclusion of ratings for disgust, anger, and empathy in the TUNA database and the apparent lack of subsequent analysis of these data. The aim of the present manuscript was twofold. First, present the TUNA database, and second, highlight the need to differentiate univalence, neutrality, and ambivalence. The addition of anger, disgust, and empathy aligns with the first aim and was based on two aspects. First, anger, disgust, and empathy have been shown to be relevant in the food context (Carfora et al., 2021; Kunst & Hohle, 2018; Kunst & Palacios Haugestad, 2016) and second, anger and disgust have been shown to be correlates of ambivalence towards meat (Buttlar et al., 2023; Buttlar & Walther, 2022). However, because our main aim was to generally demonstrate the pitfalls of selecting stimuli based on bipolar valence ratings, for which the three scales are not directly relevant, we report these ratings in the supplemental material (see validation tables https://osf.io/645jk/?view_only=36b00982d02a41ccb9aa010088275b7b). To eliminate confusion, we now include a section that explains the design choices that aim to make the TUNA database more versatile (see also our response to your comment R2.1.2.; Page 7 – 8, Line 156 - 172).

> *""We took several measures to maximize the usability of the TUNA database. First, we aimed to keep the TUNA database compatible with previous databases. Therefore, we oriented our validation on the highly cited food-pics [47] and the FRIDa [32]*

*databases and assessed similar validation ratings including, for example, recognizability, familiarity, desire to eat, palatability, valence, arousal, and complexity. Furthermore and as other databases that include food pictures, the validation tables for the TUNA database include nutritional information for the food images (e.g., [47]) and information about image properties (i.e., color, size, intensity, contrast, complexity; [47]). Second, the TUNA database includes ratings on anger, disgust, and empathy. These affective measures have been shown to be relevant for food consumption [48–50]. Additionally, anger and disgust are correlates of ambivalence toward meat [51,52]. For an analysis of how validation ratings relate, see Buttlar et al. [53]. Third, the database encompasses food pictures in various forms (e.g., raw vs. cooked, non-processed vs. highly processed) because processing has been shown to influence the evaluation of food [48,49]. Fourth, besides the total sample, the validation ratings can be used independent for the representative US and the German student sample. Because research has shown that validation ratings can differ depending on gender [9,47] or diet [47], the validation ratings can also be retrieved for female, male, veg*ans (i.e., vegetarians and vegans) and omnivores separately."*

R2.2.5. -Why were the participants classified into vegetarian and non-vegetarian if no analysis was performed on these data? Probably, the vegetarian/non-vegetarian condition has affected the ambivalence/univalence ratings (especially for meat). This information should be added to the results section.

Thank you for this question. As you proposed, research demonstrated that omnivores experience higher ambivalence towards meat than vegetarians (e.g., Buttlar & Walther, 2018). Therefore, we included the validation tables for the different subsamples. While this might only be important for some specific research questions, it was not relevant for our demonstration of the need to distinguish between ambivalence, neutrality and univalence. However, due to the classification within the TUNA desktop app, researchers can select stimuli based on the validation ratings from either one group if necessary. Please not that we provided separate ratings for women vs. men, as well as German student sample vs. representative US following the same logic. By providing these separate validation tables, we hope to increase the usability of the database. To explain this reasoning, we added a sentence within the manuscript (Page 8, Line 170 – 172; see also our response to your previous comment R2.2.4.).
